# *PIK3R3* is a candidate regulator of platelet count in people of Bangladeshi ancestry

**DOI:** 10.1016/j.rpth.2023.100175

**Published:** 2023-05-14

**Authors:** Kate Burley, Lucy Fitzgibbon, David van Heel, Genes & Health Research Team@EastLondonGenes, Shaheen Akhtar, Mohammad Anwar, Elena Arciero, Omar Asgar, Samina Ashraf, Gerome Breen, Raymond Chung, Charles J. Curtis, Shabana Chaudhary, Maharun Chowdhury, Grainne Colligan, Panos Deloukas, Ceri Durham, Faiza Durrani, Fabiola Eto, Sarah Finer, Ana Angel Garcia, Chris Griffiths, Joanne Harvey, Teng Heng, Qin Qin Huang, Matt Hurles, Karen A. Hunt, Shapna Hussain, Kamrul Islam, Ben Jacobs, Ahsan Khan, Amara Khan, Cath Lavery, Sang Hyuck Lee, Robin Lerner, Daniel MacArthur, Daniel Malawsky, Hilary Martin, Dan Mason, Mohammed Bodrul Mazid, John McDermott, Sanam McSweeney, Shefa Miah, Sabrina Munir, Bill Newman, Elizabeth Owor, Asma Qureshi, Samiha Rahman, Nishat Safa, John Solly, Farah Tahmasebi, Richard C. Trembath, Karen Tricker, Nasir Uddin, David A. van Heel, Caroline Winckley, John Wright, Dragana Vuckovic, Andrew D. Mumford

**Affiliations:** 1 School of Physiology, Pharmacology and Neuroscience, University of Bristol, Bristol, UK; 2 School of Cellular and Molecular Medicine, University of Bristol, Bristol, UK; 3 Blizard Institute, Barts and the London School of Medicine and Dentistry, Queen Mary University of London, London, UK; 4 Wolfson Institute of Population Health, Barts and the London School of Medicine and Dentistry, Queen Mary University of London, London, UK; 5 Department of Epidemiology and Biostatistics, School of Public Health, Faculty of Medicine, Imperial College London, London, UK

**Keywords:** Bangladesh, blood platelets, cardiovascular diseases, genome-wide association study, phosphatidylinositol 3-kinases

## Abstract

**Background:**

Blood platelets are mediators of atherothrombotic disease and are regulated by complex sets of genes. Association studies in European ancestry populations have already detected informative platelet regulatory loci. Studies in other ancestries can potentially reveal new associations because of different allele frequencies, linkage structures, and variant effects.

**Objectives:**

To reveal new regulatory genes for platelet count (PLT).

**Methods:**

Genome-wide association studies (GWAS) were performed in 20,218 Bangladeshi and 9198 Pakistani individuals from the Genes & Health study. Loci significantly associated with PLT underwent fine-mapping to identify candidate genes.

**Results:**

Of 1588 significantly associated variants (*P* < 5 × 10^−8^) at 20 loci in the Bangladeshi analysis, most replicated findings in prior transancestry GWAS and in the Pakistani analysis. However, the Bangladeshi locus defined by rs946528 (chr1:46019890) did not associate with PLT in the Pakistani analysis but was in the same linkage disequilibrium block (*r*^2^ ≥ 0.5) as PLT-associated variants in prior East Asian GWAS. The single independent association signal was refined to a 95% credible set of 343 variants spanning 8 coding genes. Functional annotation, mapping to megakaryocyte regulatory regions, and colocalization with blood expression quantitative trait loci identified the likely mediator of the PLT phenotype to be *PIK3R3* encoding a regulator of phosphoinositol 3-kinase (PI3K).

**Conclusion:**

Abnormal PI3K activity in the vessel wall is already implicated in the pathogenesis of atherothrombosis. Our identification of a new association between *PIK3R3* and PLT provides further mechanistic insights into the contribution of the PI3K pathway to platelet biology.

## Essentials

• Understanding which genes control platelet count provides insights into atherothrombosis.
• To identify new platelet genes, we analyzed data from Bangladeshi and Pakistani populations.
• Most platelet genes identified in our analysis overlapped with those in other populations.
• A new region containing the *PIK3R3* gene was linked to platelet count in Bangladeshis.

## 1 Introduction

Circulating platelet count (PLT) is an independent predictor of morbidity and mortality from multiple cardiovascular and inflammatory disorders, including atherothrombosis [[1], [2], [3]], but is influenced by complex sets of interacting genes (*h*^2^ > 0.3-0.8) [4,5] that may be different between ancestries [1,6]. Understanding the genetic basis of PLT offers important insights into the pathophysiology of platelet-mediated cardiovascular disease and disparities in health outcomes between populations [7,8].

Large genetic association studies for PLT and other blood cell traits have historically been restricted to European (EUR) populations [9]. However, utilization of non-EUR populations can reveal novel genetic associations because of differences in allele frequency, linkage disequilibrium (LD) structure, and variant effects driven by environmental selection pressures and genetic drift [10]. Transancestry and ancestry–specific genome-wide association studies (GWAS) have already exploited this to reveal multiple new loci for PLT [1,6]. Here, we extend this approach by performing a GWAS in a UK collection of individuals from Bangladesh alongside a comparator population of individuals from Pakistan.

## 2 Methods

Analyses were performed on the July 2021 data release of the Genes & Health study [11] in accordance with ethical approval from the London South-East NRES Committee of the Health Research Authority (14/LO/1240). The analysis populations comprised predominantly first-generation British Bangladeshi and British Pakistani adults genotyped using the Illumina Infinium Global Screening Array v3 chip.

Phenotype data were derived from linked electronic health records, which included blood cell counts measured using a Sysmex XE-2100 analyzer (Sysmex, Kobe, Japan). PLT for each individual was defined as the mean of all PLTs recorded in the electronic health records. PLT were adjusted for sex, age, height, and weight, and rank-based inverse normal transformation was applied. Associations between PLT and variants imputed using the TOPMed-r2Minimac4 1.5.7 Imputation Server were calculated using BOLT-LMM v2.3.6 using the first 10 genetic principal components as covariates. Index variants were defined as those with the lowest *P* value within a genome-wide significant (*P* < 5 × 10^−8^) locus. Conditional analyses were performed at each significantly associated locus using the index variant as a covariate to detect the presence of secondary association signals. The contributions of associated variants from each locus to PLT were evaluated further by comparing allele frequencies and effect sizes between the Bangladeshi and Pakistani populations. We tested for colocalization of variants in genomic regions associated with PLT in previous transancestry GWAS and in relevant subpopulations [1] to detect whether the Bangladeshi index variants were likely to represent the same or different association signals. Variants in the Bangladeshi association interval defined by the index variant rs946528 underwent functional annotation using Ensembl Variant Effect Predictor (VEP) to identify their likely effect on coding genes, mapping to epigenetic data from megakaryocytes, and colocalization analysis with whole blood expression quantitative trait loci (eQTLs) (Supplementary Methods).

## 3 Results and Discussion

The characteristics of the 20,218 Bangladeshi and 9198 Pakistani individuals in the analysis populations are summarized in Supplementary Table S1. The mean PLT was lower in the Bangladeshi (mean, 266.4 × 10^9^/L) than in the Pakistani populations (271.5 × 10^9^/L; *P* = 1.16 × 10^−10^; Supplementary Fig. S1). Although small case series have shown that the frequency of thrombocytopenia (PLT <150 × 10^9^/L) is higher in residents of the Eastern Indian subcontinent compared to other regions [12], we were unable to detect an increase in thrombocytopenia in British Bangladeshis (Supplementary Table S1). Single nucleotide polymorphism (SNP)-based heritability for PLT was 26.9% in the Bangladeshis and 25.3% in Pakistanis.

**Figure 1:**
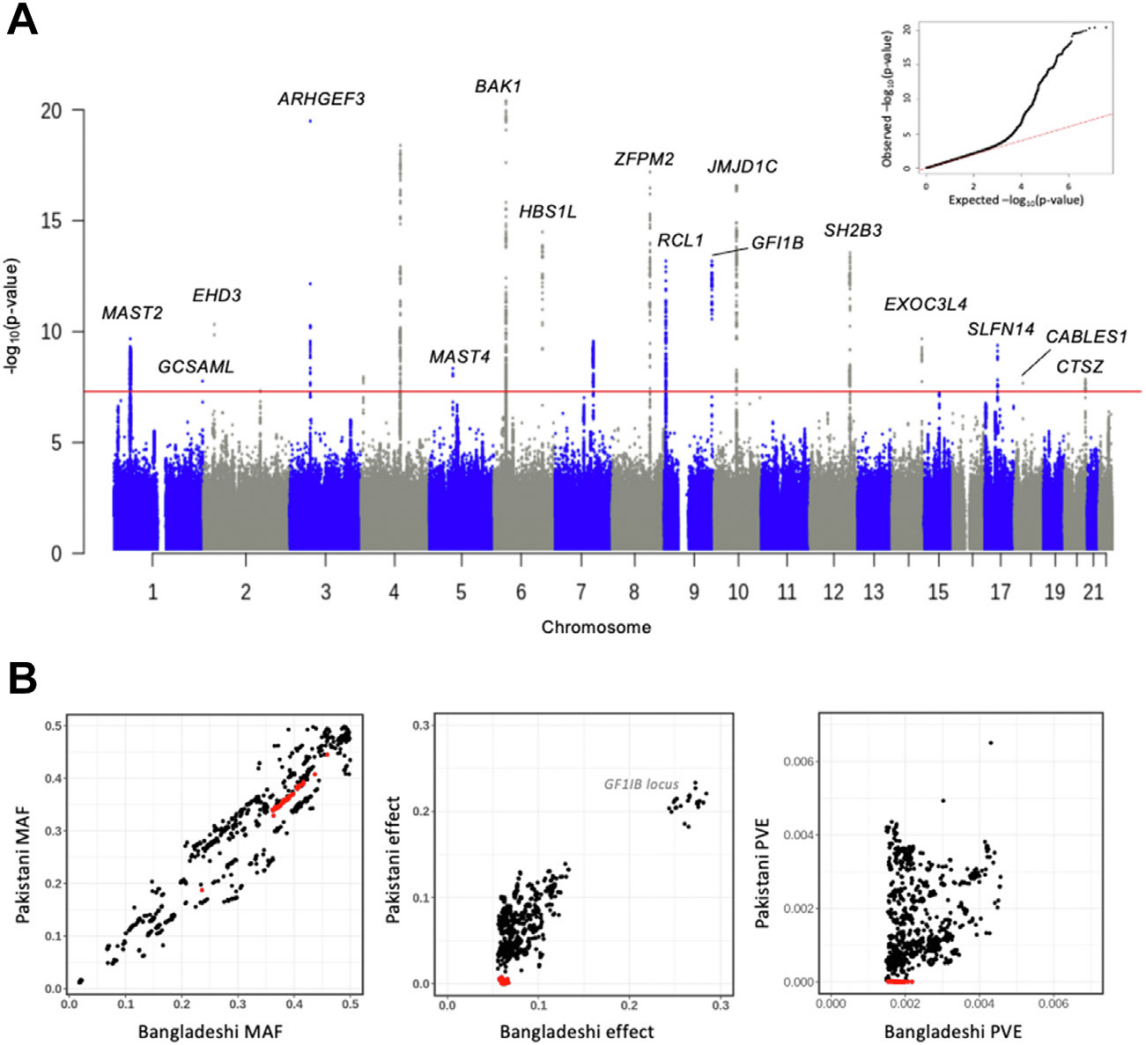
Genomic associations with platelet count in the Bangladeshi population (*n* = 20,292). (A) Manhattan plot showing 20 loci in which the index variant has a probability of association above the genome-wide significance threshold of *P* < 5 × 10^−8^ (red line). Selected loci are annotated with gene names identified using variant effect predictor most severe consequence option. The inset figure is the quantile-quantile plot of genome-wide association study *P* values. (B) Minor allele frequency (MAF), effect size (beta), and phenotypic variation explained (PVE) for each of the 1588 platelet count–associated variants identified in the Bangladeshi population compared to the same variants in the Pakistani population. For the Bangladeshi locus on chromosome 1, variants in the association interval (*r*^2^ > 0.5) of the index variant rs964528 are indicated by the red dots.

The genomic inflation factors in the Bangladeshi and Pakistani populations were 1.096 and 1.047, respectively, indicating adequate control of population stratification. In the Bangladeshi population, 1588 variants were associated with PLT at a genome-wide significance threshold of *P* < 5 × 10^−8^, assigned to 20 different loci (Figure 1, Table). All 20 Bangladeshi index variants occurred at approximately the same frequency in other ancestries in the 1000 Genomes Continental genomes dataset with the exception of rs149810016 in *GFI1B* that was present only in the South Asian (SAS) dataset (Supplementary Table S2). Conditional analysis of the variants at each locus revealed that 2 loci had secondary signals of association (*P* < 5 × 10^−8^). These were rs3846855 and rs653178, which mapped to *GGNBP1* and *ATXN2*, respectively (Supplementary Fig. S2A), both of which were associated with PLT in the transancestry or ancestry-specific GWAS of Chen et al. [1], and rs653178 was in LD with the index variant for that region.

**Table:** Platelet count–associated index variants identified in Bangladeshi individuals (*n* = 20,218) and colocalization with prior transancestry and ancestry–specific genome-wide association studies [1].

Bangladeshi index variants							Posterior probability of a common shared association signal (H_4_)		
Chromosomal position	rsID	Gene(s) with VEP most severe consequence	Coded/alternate allele	Alternate allele frequency	Beta (SE)	*P* value	Transancestry	SAS	EAS
chr1:46019890	rs946528	*MAST2*	C/T	0.58	0.067 (0.011)	2.1 × 10^−10^	2.8%	4.0%	34.7%
chr1:247549001	rs41315846	*GCSAML*	T/C	0.41	0.063 (0.011)	1.7 × 10^−8^	100.0%	-	-
chr2:31258101	rs592039	*EHD3*	G/A	0.85	0.106 (0.016)	4.7 × 10^−11^	99.7%	-	-
chr2:159926221	rs1877194	Intergenic	A/G	0.46	−0.056 (0.010)	4.7 × 10^−8^	52.6%	4.5%	11.1%
chr3:56815721	rs1354034	*ARHGEF3*	T/C	0.50	0.093 (0.010)	3.2 × 10^−20^	100.0%	-	-
chr4:6889792	rs11734132	Intergenic	G/C	0.19	0.079 (0.014)	1.1 × 10^−8^	99.6%	-	-
chr4:110027510	rs80079941	Intergenic	G/C	0.18	−0.118 (0.013)	4.0 × 10^−19^	<1%	68.5%	98.8%
chr5:66710497	rs59596869	*MAST4*	C/T	0.13	0.089 (0.015)	4.6 × 10^−9^	<1%	53.6%	9.6%
chr6:33575632	rs210139	*BAK1*	A/C	0.71	0.103 (0.011)	3.9 × 10^−21^	30.8%	95.8%	98.0%
chr6:135100038	rs34164109	*HBS1L*	C/T	0.11	0.130 (0.017)	3.2 × 10^−15^	96.7%	-	-
chr7:106700379	rs342244	Intergenic	T/G	0.35	−0.069 (0.011)	2.7 × 10^−10^	90.3%	-	-
chr8:105570896	rs4734879	*ZFPM2*	A/G	0.32	−0.103 (0.012)	6.3 × 10^−18^	99.4%	-	-
chr9:4788616	rs35797651	*RCL1*	C/G	0.35	0.084 (0.011)	6.5 × 10^−14^	96.1%	-	-
chr9:132987359	rs149810016	*GFI1B*	C/A	0.02	−0.284 (0.038)	6.6 × 10^−14^	<1%	<1%	6.2%
chr10:63267383	rs7098181	*JMJD1C*	G/T	0.48	0.085 (0.010)	2.6 × 10^−17^	98.6%	-	-
chr12:111411711	rs7309325	*SH2B*	G/T	0.41	0.077 (0.010)	2.8 × 10^−14^	<1%	97.2%	88.9%
chr14:103098397	rs61007561	*EXOC3L*	A/AG	0.29	0.072 (0.011)	2.1 × 10^−10^	98.1%	-	-
chr17:35563315	rs55910622	*SLFN14*	G/T	0.07	0.124 (0.020)	4.2 × 10^−10^	57.3%	97.6%	0.1%
chr18:23141009	rs11082304	*CABLES1*	G/T	0.46	−0.057 (0.010)	2.1 × 10^−8^	100.0%	-	-
chr20:58999408	rs163787	*CTSZ*	A/G	0.80	0.076 (0.013)	1.4 × 10^−8^	<1%	97.7%	0.0%

Bangladeshi index variants were defined as those with the lowest *P* value within each associated locus. Chromosomal positions are expressed relative to the GRCh38 genome assembly with the coded/alternate alleles on the positive strand. Genes were assigned to each index variant by annotating with VEP and selecting the gene with the most severe functional consequence. Effect size (beta), SE, and probability of association are shown for each Bangladeshi index variant. Data are also presented for a colocalization analysis generated using the coloc( ) R package in which the platelet count associations for all variants within 500 kB of the Bangladeshi index variants were compared in the transancestry genome-wide association study population. If there was a low posterior probability of colocalization (<80%), then colocalization was tested against the SAS and EAS genome-wide association study analysis population [1].

EAS, East Asian; SAS, South Asian; VEP, variant effect predictor.

In the smaller Pakistani population, there were 68 PLT-associated variants at 5 loci (Supplementary Fig. S3, Supplementary Table S3). The allele frequencies, effect sizes, and phenotypic variation explained (PVE; the contribution that each variant makes to the spread of PLT in the population) of the PLT-associated variants in the Bangladeshi population were in most cases similar to those in the Pakistani population. However, for the Bangladeshi index variant rs946528 (chr1:46019890), variants in LD with the index variant (defined as *r*^2^ ≥0.5) were conspicuous because of a disproportionately greater effect size on PLT in the Bangladeshi compared with the Pakistani population, resulting in greater PVE. Allele frequencies between the Bangladeshi and Pakistani study populations were similar (Figure 1B).

In order to explore whether the PLT loci identified in the Bangladeshi GWAS replicated associations in the largest-to-date transancestry meta-GWAS of blood cell traits comprising 721,201 individuals [1], we performed colocalization analyses for sets of variants within 500 kB of each of the 20 Bangladeshi index variants (Table, Supplementary Table S6). For 11 of the Bangladeshi index variants, there was a high posterior probability (PP) that the association signal with PLT in the transethnic GWAS was driven by the same variant (PP of H_4_ >0.8). A further 5 variants had a similarly high likelihood of colocalization with PLT-associated variants in either the SAS or East Asian (EAS) ancestry–specific GWAS populations [1] (Table and Supplementary Table S6).

Of the remaining 4 Bangladeshi index variants that did not colocalize with previous PLT-associated variants, rs149810016 and rs59596869 mapped by VEP to *GFI1B* and to *MAST4*, respectively, which are PLT-associated genes identified from other index variants in the meta-GWAS by Chen et al. [1] rs1877194 was annotated by VEP to an intergenic region but is adjacent to the PLT-associated *LY75-C302*. These findings suggest that these association signals potentially indicate new regulatory mechanisms for PLT mediated through their respective annotated genes. The final remaining Bangladeshi locus with index variant rs946528 uniquely showed a single association signal with PLT (Supplementary Fig. S2B), no colocalization with PLT-associated variants in the transancestry and EAS or SAS GWAS (Table, Supplementary Table S6) and was not annotated to PLT genes identified in the meta-GWAS.

**Figure 2:**
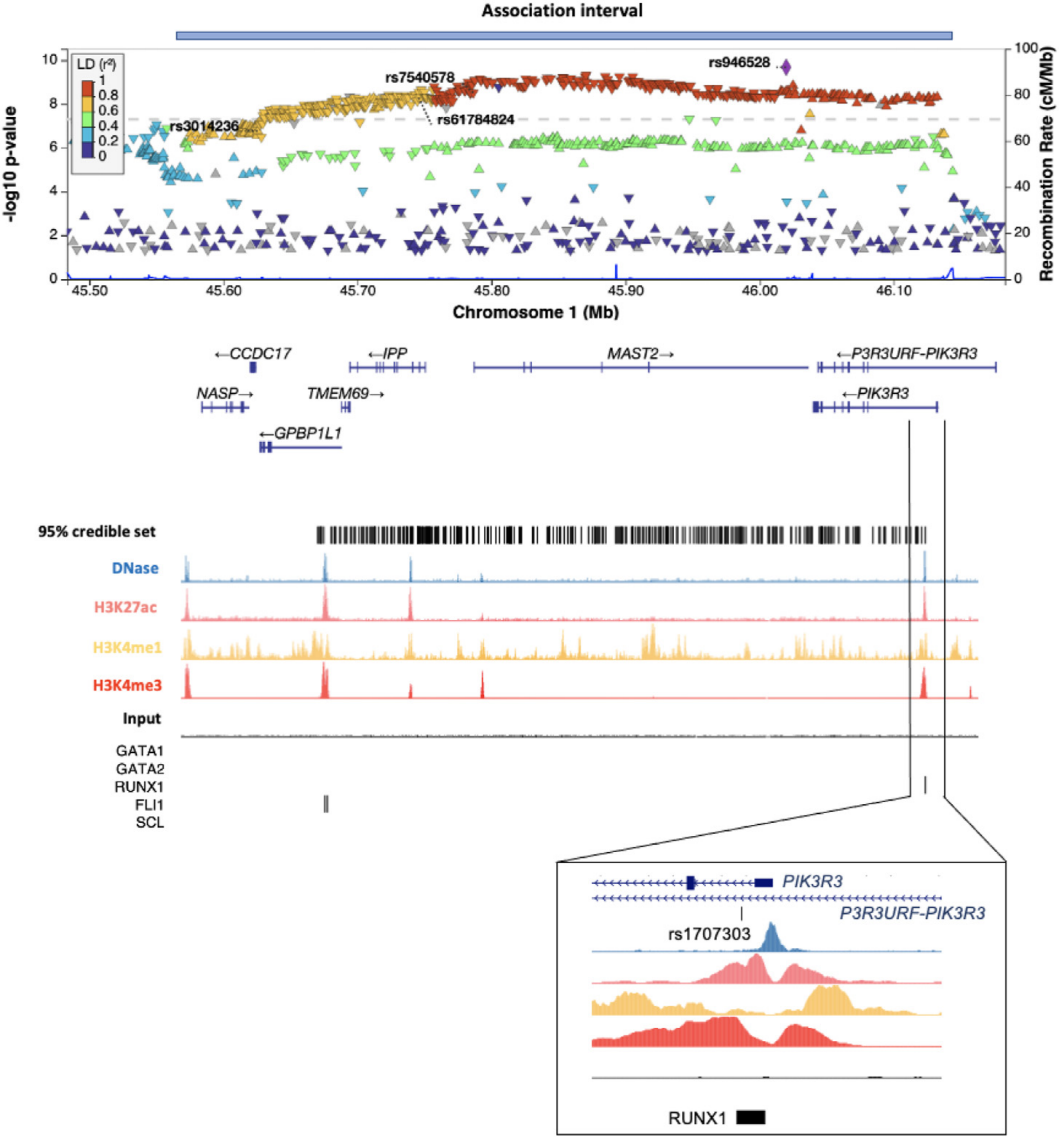
Detailed view of the Bangladeshi rs946528 association interval. LocusZoom plot of associations in the 562 kB association interval for the chromosome 1 index variant rs946528, defined as variants within *r*^2^ ≥ 0.5 calculated using linkage disequilibrium (LD) reference data from the Bengali from Bangladesh 1000 Genomes dataset. Variants with *P* > .05 are excluded to enable visualization of the recombination peaks. Indicated variants are the index rs946528 and the 3 variants associated with platelet count in prior East Asian ancestry–specific genome-wide association studies. Beneath and aligned with this are the 8 UCSC Genome Browser–annotated genes within the interval; positions of the 95% credible set of 343 variants and annotated regions of epigenetic activity from CD34^−^, CD41^+^, and CD42^+^ megakaryocytes, the progenitor cell for circulating platelets, which were provided by the BLUEPRINT Epigenomics Project [25]. Megakaryocyte chromatin immunoprecipitation sequencing (ChIP-seq) data are also shown indicating relevant transcription factor binding sites [16]. The expanded view shows the relationship between the significantly associated variant rs1707303 and a putative regulatory region surrounding the first exon of the candidate gene *PIK3R3.*

The locus containing Bangladeshi index variant rs946528 comprised a 562 kB region (chr1:45575428-46137676) of low recombination containing a 95% credible set of 343 variants (Figure 2). This region did contain other variants associated with PLT in the EAS ancestry–specific GWAS performed by Chen et al. [1] and 2 other NHGRI-EBI–cataloged GWAS that were restricted to EAS populations (Supplementary Figure S4, Supplementary Table S4) [13,14]. All of these variants were >200 kB from rs946528 and since none colocalized with variants in the rs946528 region, this likely represents a novel PLT-associated signal.

Variants in LD (*r*^2^ > 0.5) with rs946528 span 8 UCSC Genome Browser–annotated genes (Figure 2, Supplementary Table S5). Among the genes contained within this region, only *PIK3R3* was represented in both the platelet transcriptome and proteome catalogs within the PlateletWeb functional genomics database [15]. Moreover, among all variants in the 95% credible set, only rs1707303 in intron 1 of *PIK3R3* overlapped with a prominent area of epigenetic activity, suggesting that this variant lies within a regulatory region (Figure 2). At least part of this epigenetic activity may be accounted for by the immediate adjacency of rs1707303 to a consensus binding site for RUNX1, a critical transcription factor necessary for differentiation and maturation of platelet-forming megakaryocytes [16]. The rs946528 association signal colocalized with cis-eQTLs in whole blood for *MAST2* and *IPP* with posterior probabilities of 45.2% and 31.4%, respectively, below the threshold of 80% usually considered indicative of a shared causal variant (Supplementary Fig. S5, Supplementary Table S5). Neither *MAST2* nor *IPP* are expressed in platelets and neither has a plausible biological role in platelet production [15].

In this first reported GWAS for a hematological trait in a Bangladeshi population, several lines of evidence suggested that the chromosome 1 locus defined by the index variant rs946528 represented a novel association signal with PLT. First, the rs946528 haplotype had disproportionately larger PVE estimates in the Bangladeshi than in the Pakistani population, for which data collection and analysis methods were identical. This finding was driven by differences in variant effect size between the Bangladeshi and Pakistani populations and not by minor allele frequency. Although variants within this locus also drove an association with PLT in a large prior transancestry and EAS–specific GWAS [1], colocalization analysis suggested that this was through different causal variants and that the driver variant in the Bangladeshi population was in a distant genomic location and likely linked to a different gene. It was noteworthy that the Bangladeshi rs946528 association window also included variants that replicated findings from other GWAS for PLT in previous EAS populations but which have not been previously annotated [1,13,14]. By contrast, the other PLT-associated loci in the Bangladeshi and Pakistani GWAS replicated prior transancestry GWAS, and in most cases, they were linked to genes already implicated in platelet biology [1]. Replication of the rs946528 association interval as an apparently ancestral EAS PLT-associated locus was unsurprising given that modern Bangladeshi populations are predominantly of SAS ancestry but with significant EAS and South-East Asian admixture [17]. This confirmatory discovery in the Bangladeshi population highlights the value of ancestry-specific GWAS in providing additional insights into the architecture of complex loci associated with population traits that complement transancestry or large EUR population GWAS [1,9].

**Figure 3:**
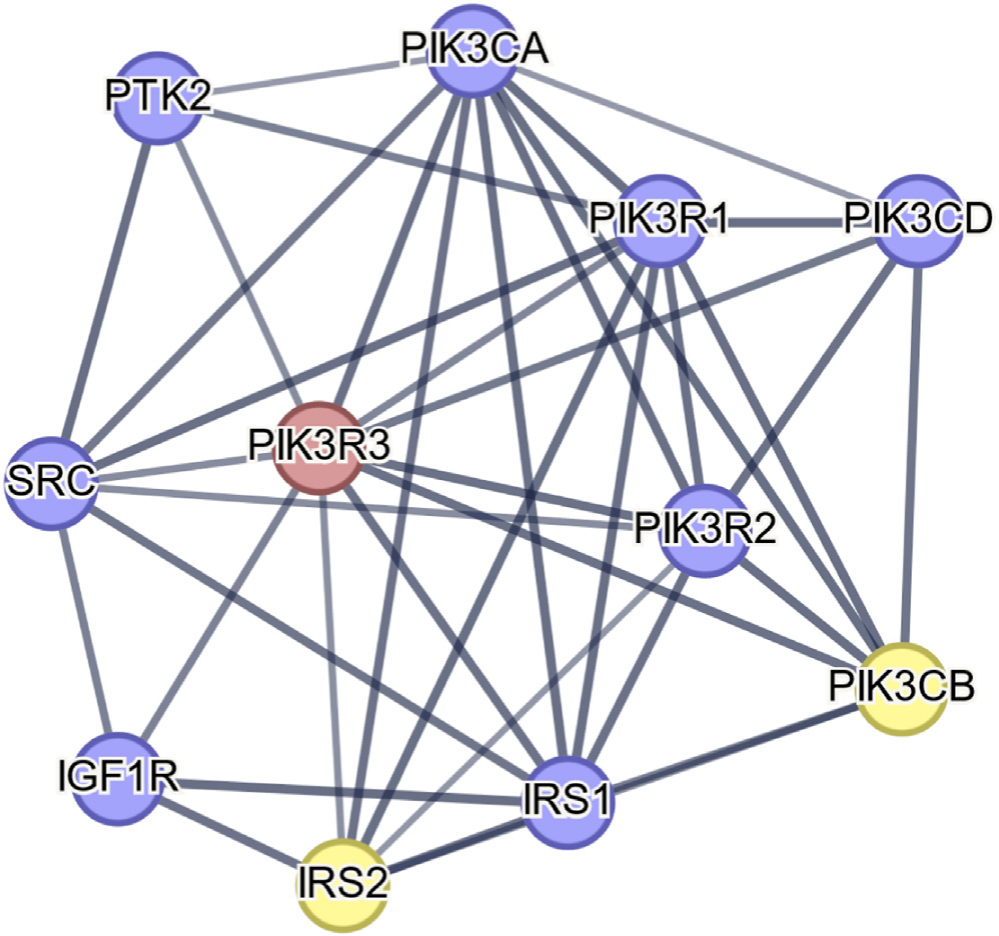
Interaction network for *PIK3R3.* The nodes represent *PIK3R3* and interacting genes designated within Platelet Web [25] as present in the platelet proteome (blue) and in both the proteome and transcriptome (yellow). The edges indicate physical interactions between the encoded proteins. Only STRING database v11.5 first order interactions with confidence scores of >0.7 are shown [19].

One significant challenge with the rs946528 locus is that the single independent association signal for PLT was attributable to 95% credible set of 343 variants in a haplotype block containing 8 annotated genes. Considering orthogonal evidence from several sources, *PIK3R3* was identified as the most likely candidate mediator of the PLT phenotype. This was primarily because the *PIK3R3* intron 1 variant rs1707303 was unique within the rs946528 haplotype in that it mapped to an area with multiple epigenetic signals indicating a *PIK3R3* regulatory region most likely related to a consensus binding site for RUNX1, a critical megakaryocyte transcription factor [16]. *PIK3R3* is further supported as a candidate mediator of the PLT phenotype because it was the only gene at this locus to be expressed within platelets and because it encodes phosphatidylinositol 3-kinase regulatory subunit gamma (PIK3R3; Uniprot Q92569). In an interactome analysis using the STRING database [18], PIK3R3 has 10 first order interactions with high confidence (>0.7 score), all with proteins that are also represented in the platelet proteome (Figure 3) [18]. The PIK3R3 interactors are enriched for proteins with the biological process Gene Ontology terms GO:0014065 phosphatidylinositol 3-kinase signaling (strength, 2.54; false discovery rate, 1.04 × 10^−13^) and GO:0048009 insulin-like growth factor receptor signaling pathway (strength, 2.61; false discovery rate, 8.08 × 10^−6^) [19], both implicated in mediating megakaryocyte maturation and platelet production [[20], [21]].

Potential limitations of this study are that the association between the rs946528 locus and PLT has not been replicated in an exclusively Bangladeshi population and that the datasets used for variant imputation and colocalization analyses have necessarily been derived from EUR populations. However, despite this, the otherwise compelling evidence that *PIK3R3* is a candidate mediator of PLT remains significant as the encoded protein PIK3R is also a mediator of vascular smooth muscle proliferation and neointimal formation in atherogenesis [22,23], which are potentially tractable to therapies that target the PI3K pathway [24]. Complementary functional characterization of the *PIK3R3* locus and testing associations with cardiovascular disease outcomes are required to complete evaluation of this locus. The GWAS summary statistics (available via the NHGRI-EBI GWAS Catalog) have future value in fine mapping other loci and for development of polygenic risk scores.

## Appendix

The current members of the Genes & Health Research Team (in alphabetical order by surname) are Shaheen Akhtar, Mohammad Anwar, Elena Arciero, Omar Asgar, Samina Ashraf, Gerome Breen, Raymond Chung, Charles J. Curtis, Shabana Chaudhary, Maharun Chowdhury, Grainne Colligan, Panos Deloukas, Ceri Durham, Faiza Durrani, Fabiola Eto, Sarah Finer, Ana Angel Garcia, Chris Griffiths, Joanne Harvey, Teng Heng, Qin Qin Huang, Matt Hurles, Karen A. Hunt, Shapna Hussain, Kamrul Islam, Ben Jacobs, Ahsan Khan, Amara Khan, Cath Lavery, Sang Hyuck Lee, Robin Lerner, Daniel MacArthur, Daniel Malawsky, Hilary Martin, Dan Mason, Mohammed Bodrul Mazid, John McDermott, Sanam McSweeney, Shefa Miah, Sabrina Munir, Bill Newman, Elizabeth Owor, Asma Qureshi, Samiha Rahman, Nishat Safa, John Solly, Farah Tahmasebi, Richard C. Trembath, Karen Tricker, Nasir Uddin, David A. van Heel, Caroline Winckley, and John Wright.
