# Supplementary material for: PIK3R3 is a candidate regulator of platelet count in people of Bangladeshi ancestry
Source: Res Pract Thromb Haemost. 2023 May 14;7(4):100175. doi: 10.1016/j.rpth.2023.100175 (PMC10394561; doi:10.1016/j.rpth.2023.100175)
Supplement: Supplementary methods [file mmc1.docx]

**SUPPLEMENTARY MATERIAL**

***PIK3R3* is a candidate regulator of platelet count in people of Bangladeshi ancestry. Burley *et al*.**

| **Supplementary methods** | Page 2 |
| --- | --- |
| **Figure S1** | Page 6 |
| **Figure S2** | Page 7 |
| **Figure S3** | Page 8 |
| **Figure S4** | Page 9 |
| **Figure S5** | Page 10 |
| **Table S1** | Page 11 |
| **Table S2** | Page 12 |
| **Table S3** | Page 13 |
| **Table S4** | Page 14 |
| **Table S5** | Page 15 |
| **Supplementary references** | Page 16 |
|  |  |

**For supplementary table S6 please see attached Excel spreadsheet**

**Supplementary methods**

**Genotyping, quality control and imputation**

Genotyping, quality control and imputation were performed by the Genes and Health study. Analysis was performed on the July 2021 data release, containing 44,396 individuals genotyped on the Illumina Infinium Global Screening Array-24 v3.0 BeadChip (in GRCh38). Quality control of genotyped data was undertaken in Ilumina GenomeStudio and plink v1.9, including removing variants with a low call rate (<0.99), rare variants with minor allele frequency (MAF) <0.0001, and variants that failed the Hardy–Weinberg test (*p* <1 ×10^−6^). Imputation was undertaken on the TOPMed-r2Minimac4 1.5.7 Imputation Server ^2^ and variants with imputation INFO score filter of >0.3 or MAF <0.00001 were excluded pre-GWAS.

**Principal component analysis for genetic inference of ethnicity**

Analysis was performed by Teng Heng, Wellcome Sanger Institute. Related individuals (second degree or closer) were identified using KING v2.2.4 ^3^, then principal component analysis (PCA) performed in all unrelated individuals to identify distinct Pakistani and Bangladeshi clusters. Related individuals were projected onto the same PC space to determine ancestry. Ancestry outliers and individuals with discrepant questionnaire reported ancestries were excluded from the analysis.

**GWAS**

Data preparation, GWAS and downstream analyses were performed in the Genes and Health Trusted Research Environment. Of the resulting 44,190 genotyped individuals, platelet counts (PLT) were available for 30,496 through linked electronic health records (EHRs). PLT for each individual was calculated as the mean of all recorded PLT. PLT were adjusted for age, sex, height and weight (imputed with k-Nearest Neighbour imputation where missing) using a linear regression model, outliers (>3*IQR from median) excluded, and rank-based inverse normal transformation applied to the residuals. Association statistics were calculated using BOLT-LMM v2.3.6 ^4^ with imputed SNP dosages using the first 10 PCs as covariates. A mixed model approach accounting for both relatedness and population stratification allowed more individuals to be included in the study.

Resulting GWAS associations were filtered to exclude variants with imputation INFO score <0.7 or MAF <0.005. Phenotypic effect sizes were calculated as the absolute additive change in the trait mean measured in standard deviations per allele. Phenotypic variation explained (PVE) per variant was calculated as 2(MAF)*(1-MAF)*beta^2. Chromosomal positions were expressed relative to the GRCh38 genome assembly with the coded/alternate alleles on the plus strand. Variants were annotated with rsIDs using dbSNP build 155 and gene annotations using Ensembl Variant Effect Predictor (VEP) v104.3.

Index variants were defined as those with the lowest p-value within a genome-wide significant (p <5 x10^-8^) locus. Conditional tests of association were performed for each significant locus (+/- 1kb of index variant) using SNPtest v2.5.2 ^6^. Variant-phenotype associations were conditioned upon the index variant, with repeated iterations including independently associated variants in a frequentist additive model until no associations remained (p <5 x10^-8^).

**SNP-based heritability**

SNP-based heritability was estimated using LDSC v1.0.1 ^5^ using CSA (Central/South Asian) LD scores downloaded from the Pan-UK Biobank (https://pan.ukbb.broadinstitute.org/).

**Colocalisation analysis of BAN PLT loci**

All variants within 500kB of the index variant for each of the 20 PLT associated loci identified in the Bangladeshi popualtion were tested for colocalization with variants in the same genomic regions in the transethnic meta GWAS reported by Chen *et*

*al* ^1^, with summary statistics downloaded from the NHGRI-EBI GWAS catalog https://www.ebi.ac.uk/gwas/. Colocalization was performed using Approximate Bayes Factor analysis in the R package coloc (https://cran.r-project.org/web/packages/coloc/).

For Bangladeshi loci that did not colcalise with transethnic signals (posterior probability of shared causal variant H_4_ <80%), colocalization was then performed using the South Asian (SAS) and East Asian (EAS) ancestry-specific GWAS. Note that the SAS GWAS contained 8,189 individuals predominantly from the UK Biobank study. The possibility of overlap of study subjects cannot be ruled out, however the UK Biobank SAS cohort contains <2% participants self-reporting as Bangladeshi or Pakistani (see DOI: 10.1186/s40246-022-00380-5) meaning the number of co-enrolled cases is likely very small and any possible confounding effect on the colocalization analysis minimal. Further colocalization analyses were performed using summary statistics from EAS-restricted GWAS reported by Sakaue *et al*. and Kanai *et al.* ^11, 12^

**Analysis of the rs946528 association region**

Statistical fine mapping of the rs946528 association region was performed using FINEMAP v1.3.1 ^7^. Input windows were defined as +- 500 kb from the index variant rs946528 (chr1:45,519,890-46,519,890). The number of conditionally independent signals in the window was used as prior knowledge for the maximum number of causative variants to be searched (–n-causal-snps option). The LD structure was computed from the same samples included in the GWAS analysis. 95% credible sets were defined as minimal sets of variants jointly covering at least 95% of the posterior probability of including the true causative signal.

Predicted regulatory regions were interrogated in the UCSC Genome Browser (http://genome.ucsc.edu/), using DNase I hypersensitivity, H3K27ac (active gene transcription), H3K4me1 (enhancer) and H3K4me3 (promoter) data tracks from CD34-negative, CD41-positive, CD42-positive megakaryocytes provided by the BLUEPRINT Epigenomics Project ^8^. ChIP-seq identified binding sites for haematopoietic transcription factors GATA1, GATA2, RUNX1, FLI1, and SCL in megakaryocytes were also integrated ^9^.

Colocalization of the GWAS signal with eQTL datasets was performed using the R package coloc as described previously. Whole blood summary cis-eQTL data was downloaded from the eQTLGen Consortium https://www.eqtlgen.org/ ^10^ and posterior probability for both traits (PLT versus eQTL) sharing a single causal variant (*H*_4_) calculated for *NASP, CCDC17, GPBP1L1, TMEM69, IPP, MAST2* and *PIK3R3*.


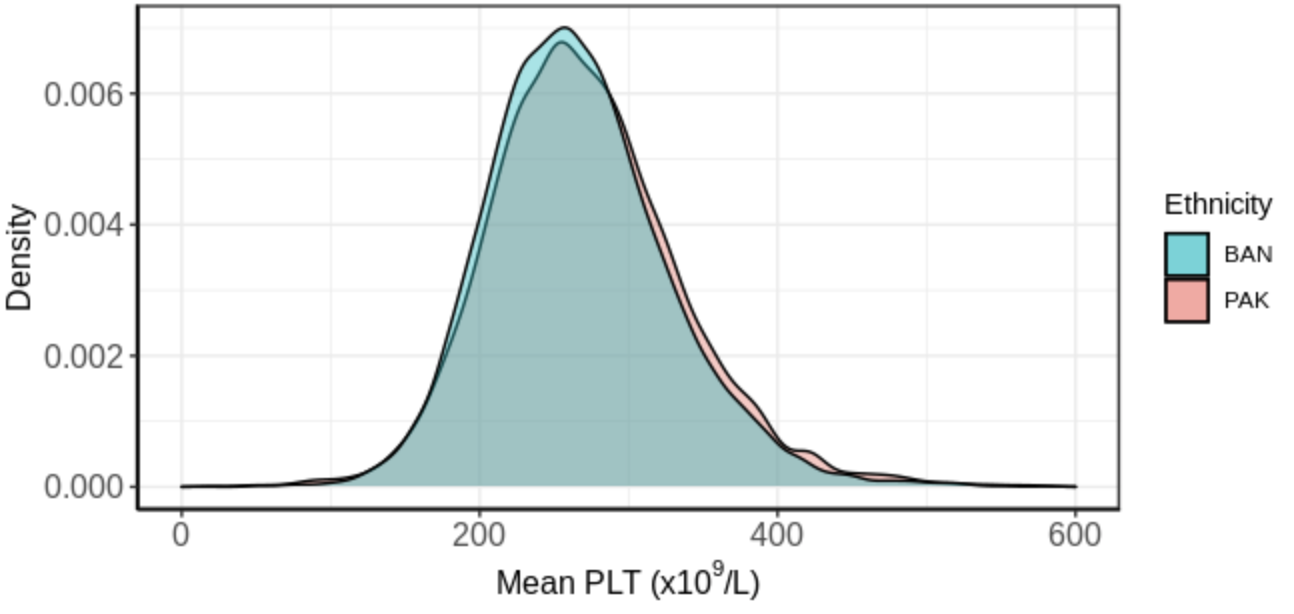


**Figure S1:** **PLT in the Bangladeshi and Pakistani populations**

For each individual in the analysis groups, the data represent the mean of all PLT documented in electronic health records within the Genes and Health dataset.

| **Chromosomal Position**  **(GRCh38)** | **rsID** | **Gene(s) with VEP most severe consequence** | **Coded/**  **alteranate allele** | **Alternate allele freq** | **Beta (SE)** | **p value** | **LD with transethnic index variant (r^2^)** |
| --- | --- | --- | --- | --- | --- | --- | --- |
| chr6:33588100 | rs3846855 | *GGNBP1* | G/A | 0.34 | 0.099 (0.011) | 5.5 x10^-21^ | 0.00 |
| chr12:111569952 | rs653178 | *ATXN2* | C/T | 0.94 | -0.092 (0.020) | 6.0 x10^-6^ | 0.57 |

**A**

**B**


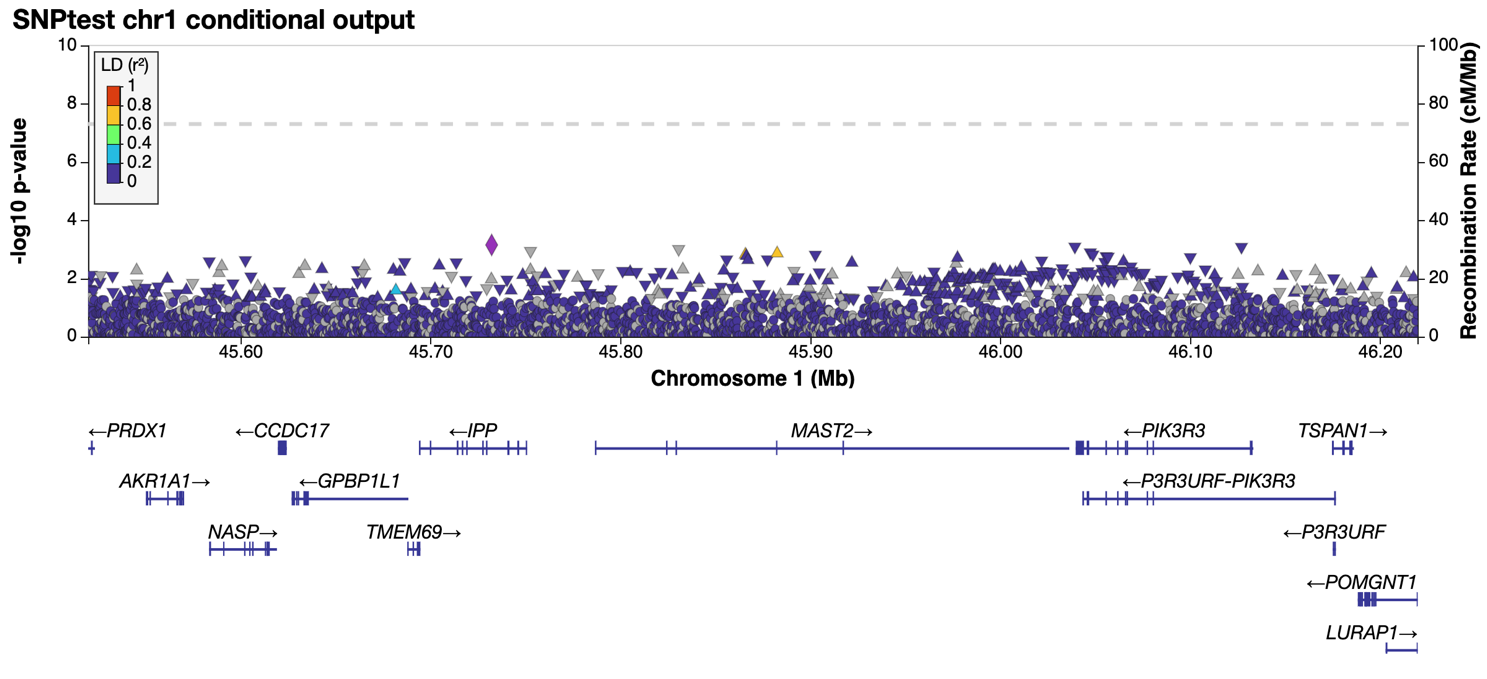


**Figure S2: Conditional analysis of genomic associations with PLT in the Bangladeshi population (n=20,292).** For each of the index varaints identified in the Bangladeshi popualtion, a conditional analysis was performed using the index variant as a covariate. **A.** Secondary signals of association were identified at the chr 6 and chr 12 loci which mapped to *GGNBP1* and *ATXN* respectively. LD is calculated using the 1000 Genomes Bengali from Bangladesh population. **B.** LocusZoom plot of the conditional analysis for the chr 1 locus containing the index variant rs946528 showing that there were no secondary signals of association in this region after conditioning on the index variant. The labelled variant (purple diamond) has the lowest p-value in the region.


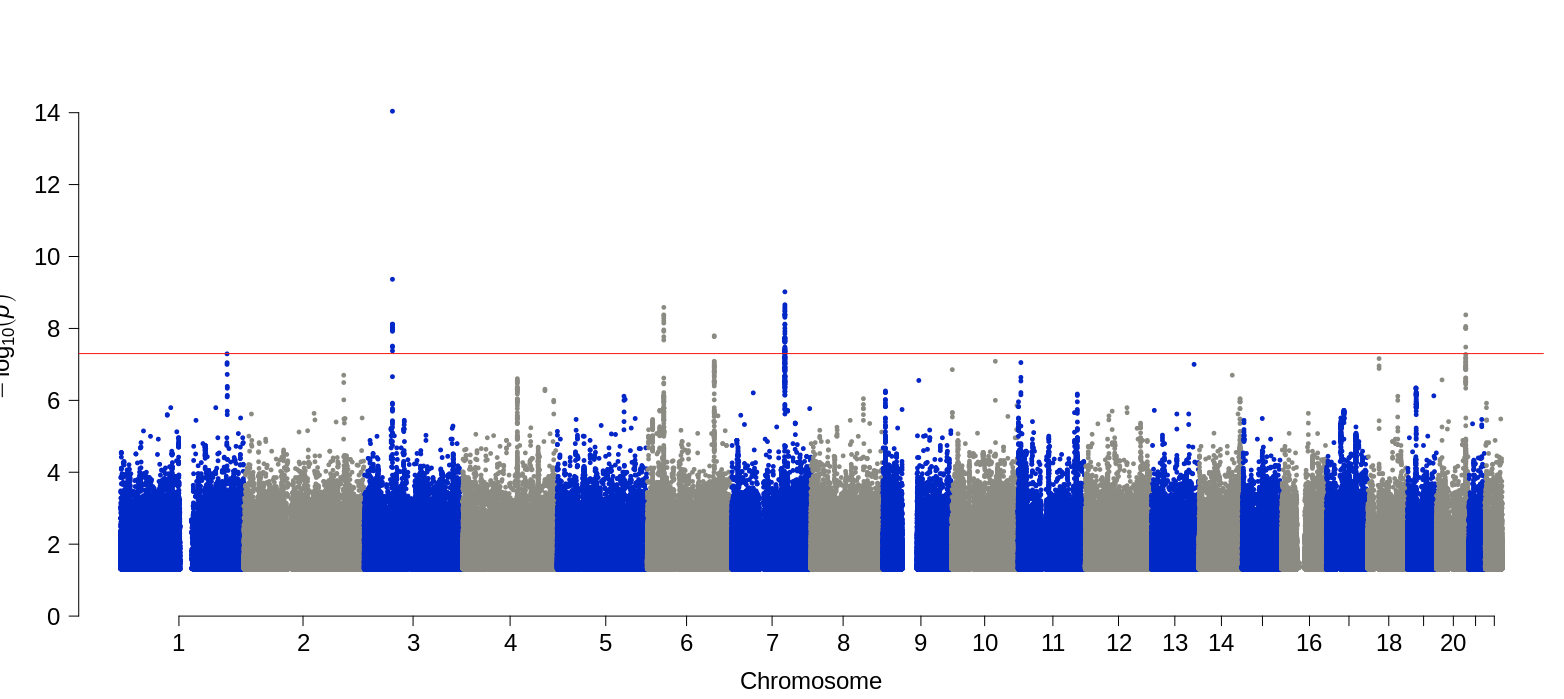

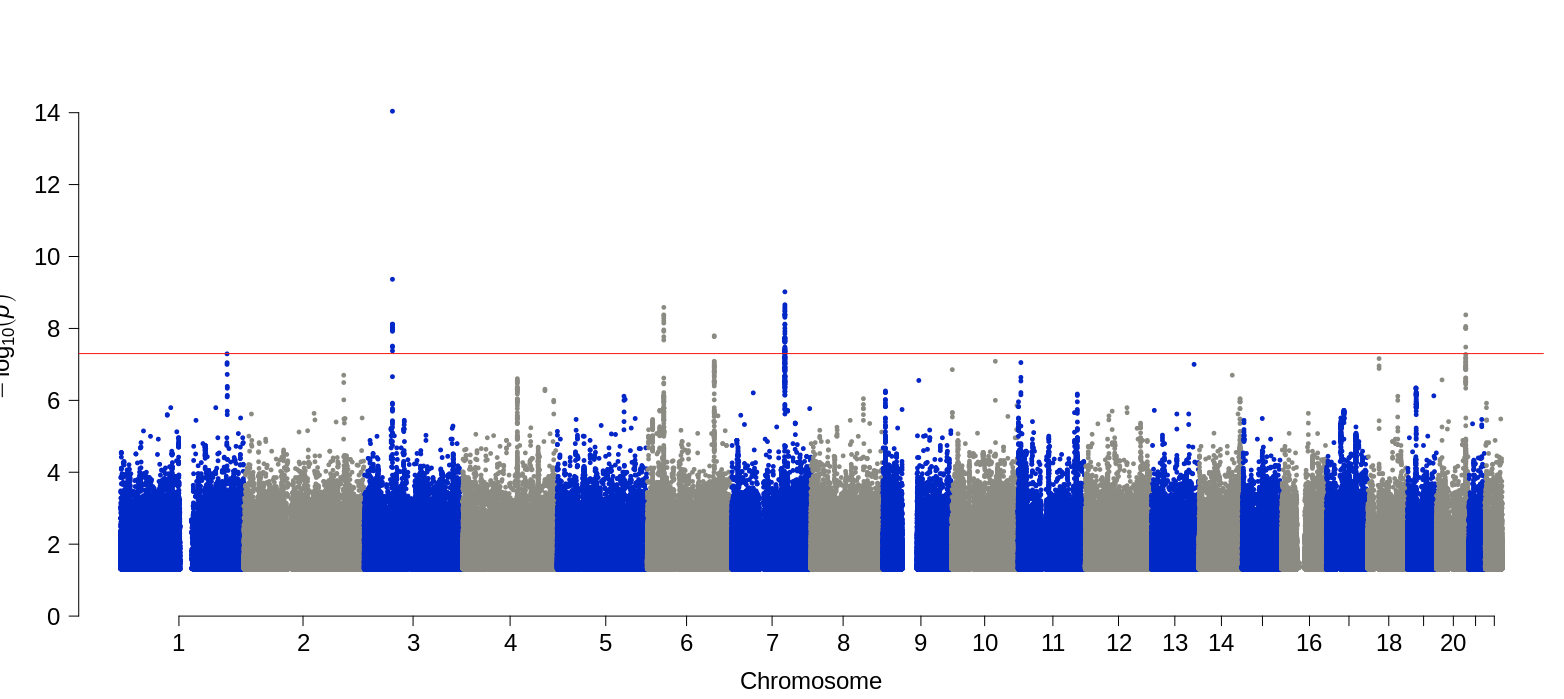

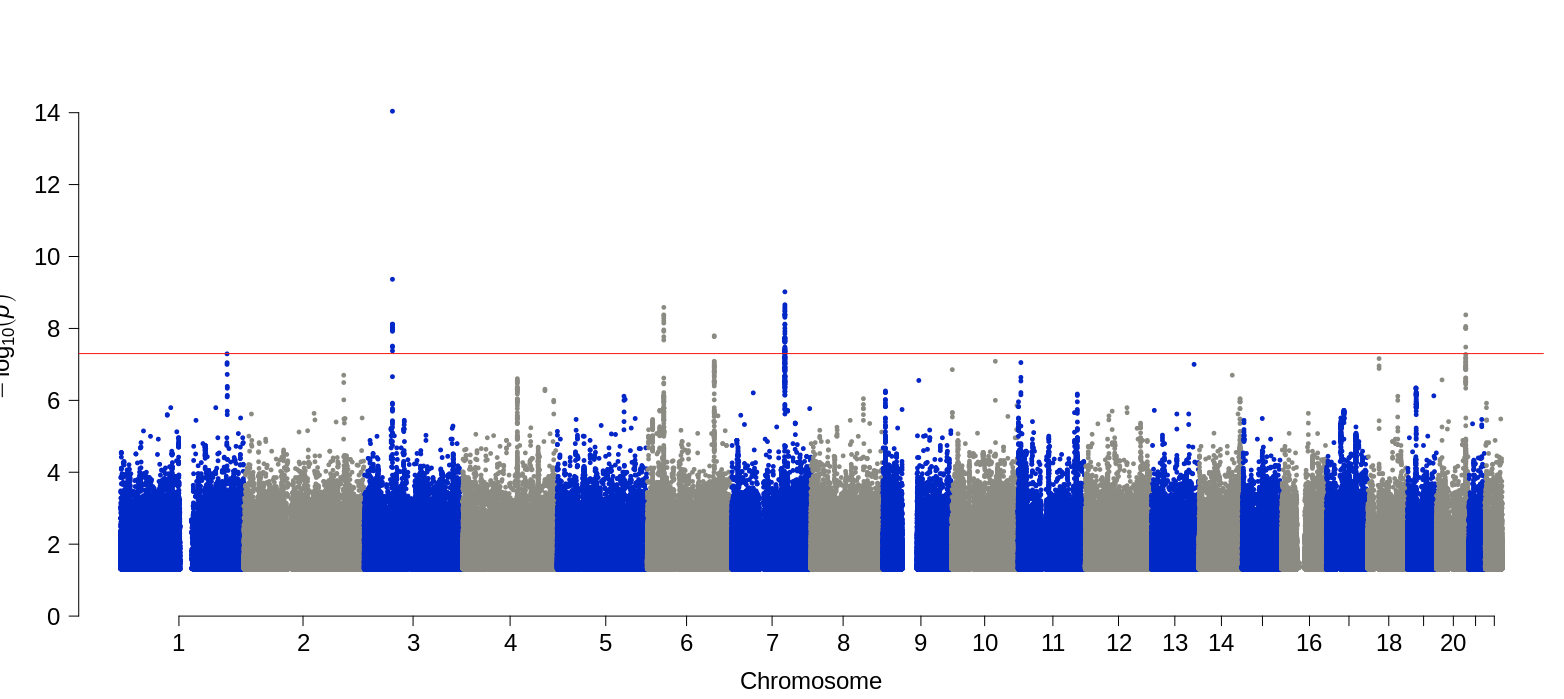

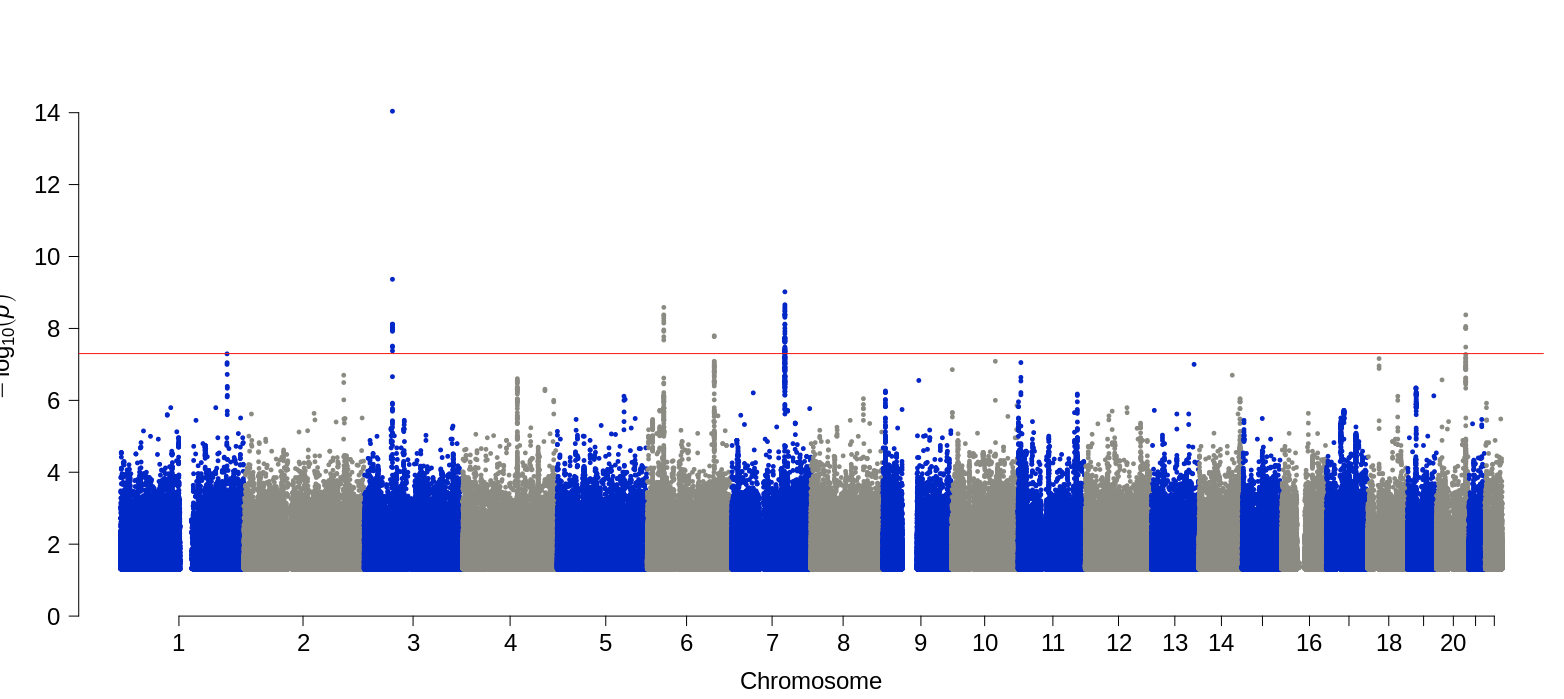


-log_10_(p-value)

Chromosome

*ARHGEF3*

*TUBB1*


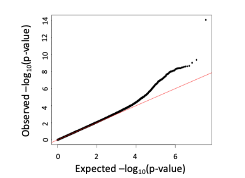


**Figure S3: Genomic associations with PLT in the Pakistani populaton (n=9198)**

**A**. Manhattan plot showing 5 loci in which the index variant has a probability of association above the genome-wide significance threshold of p <5 x10^-8^ (red line). Loci are annotated with protein-coding gene names identified using the Variant Effect Predictor (VEP) most severe consequence option (intergenic annotations not shown). The inset figure is the quantile-quantile plot of GWAS p-values.


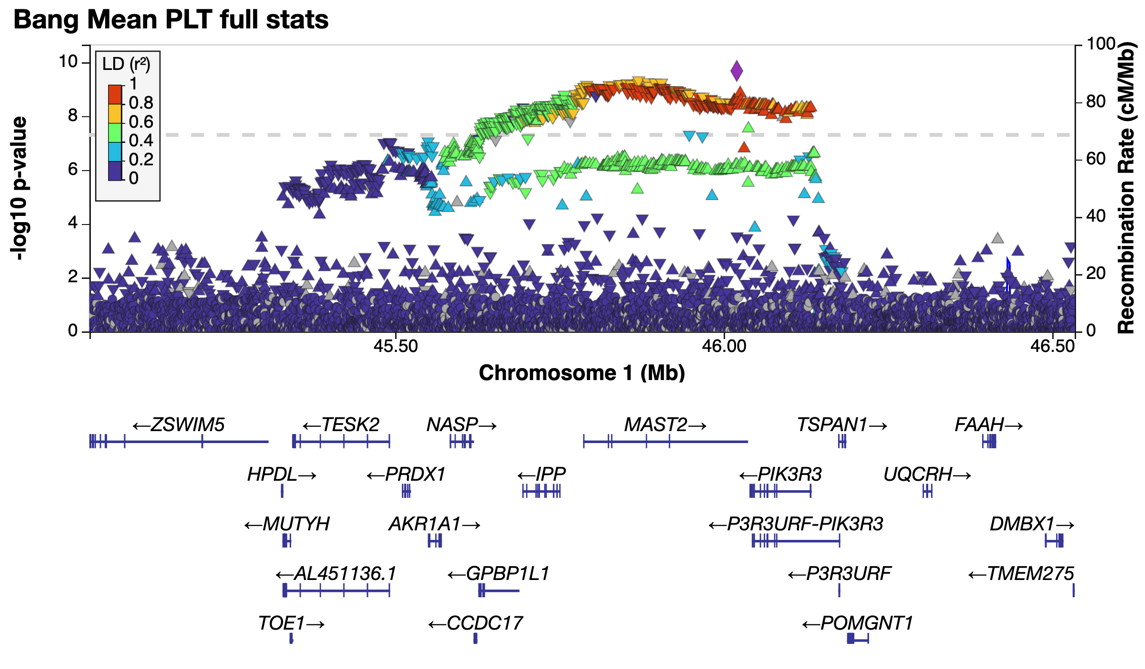


**A**


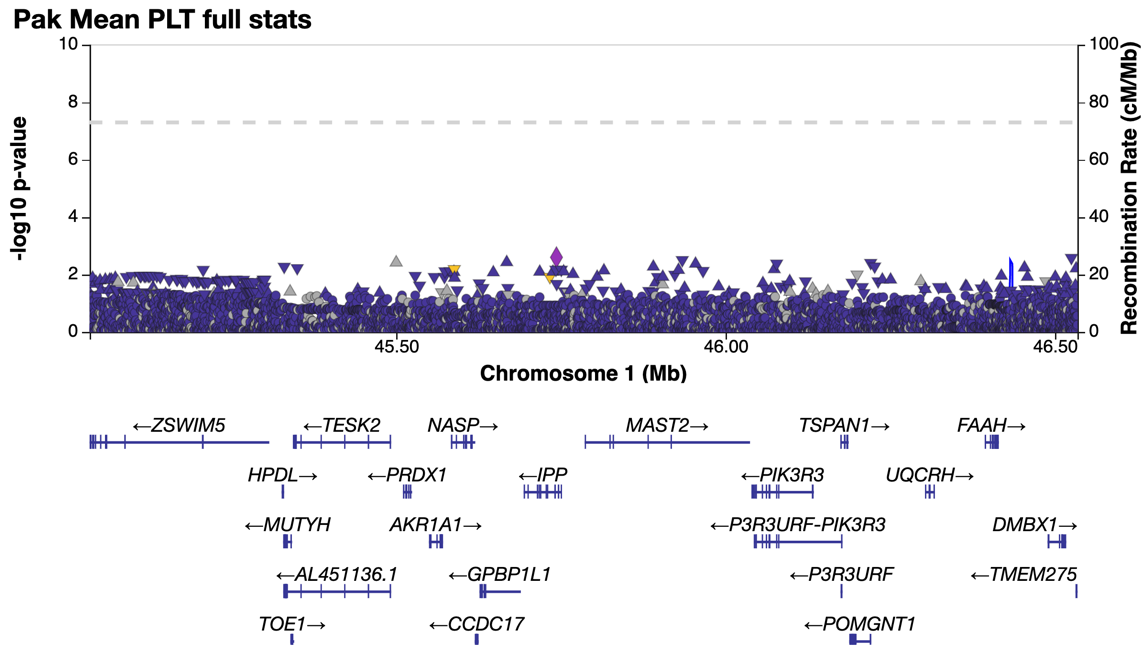

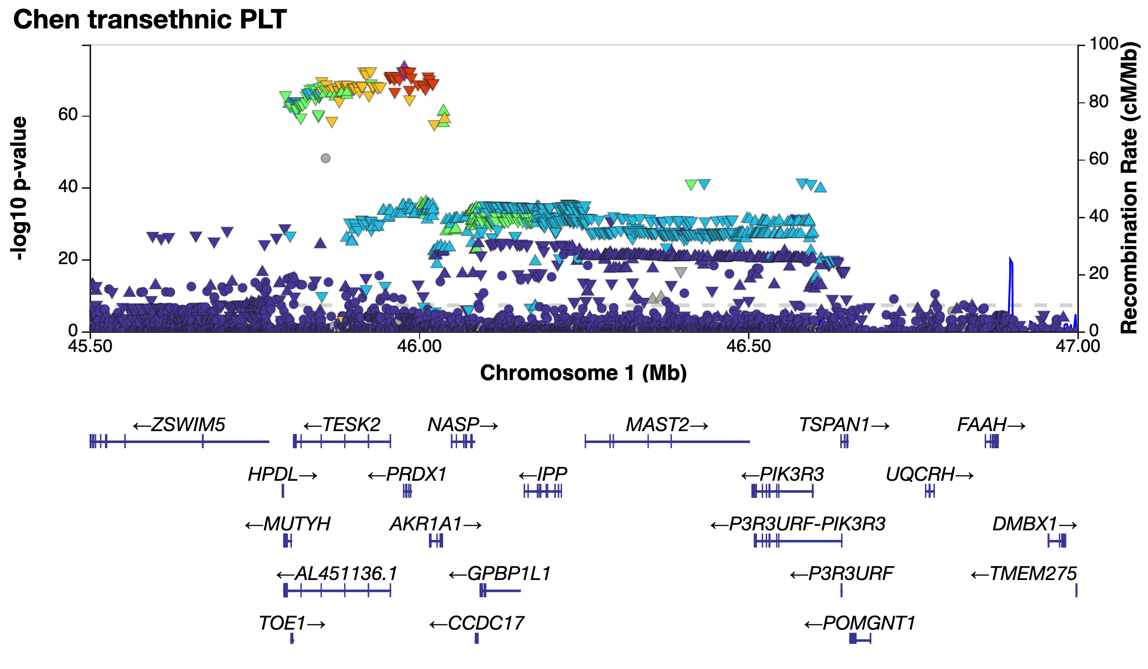

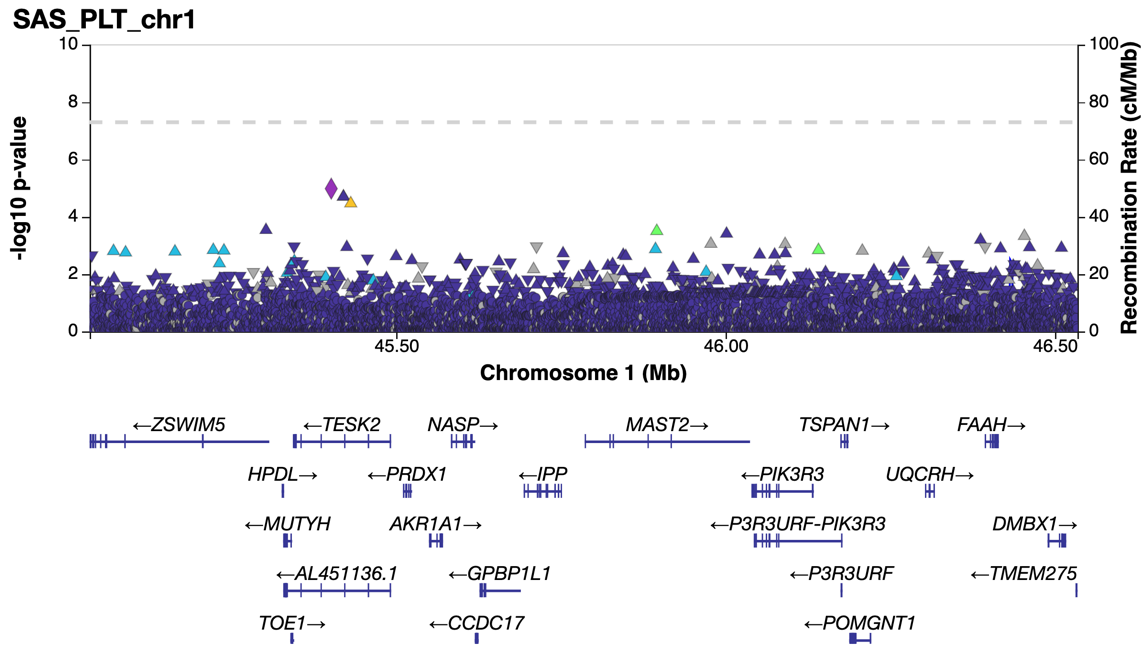

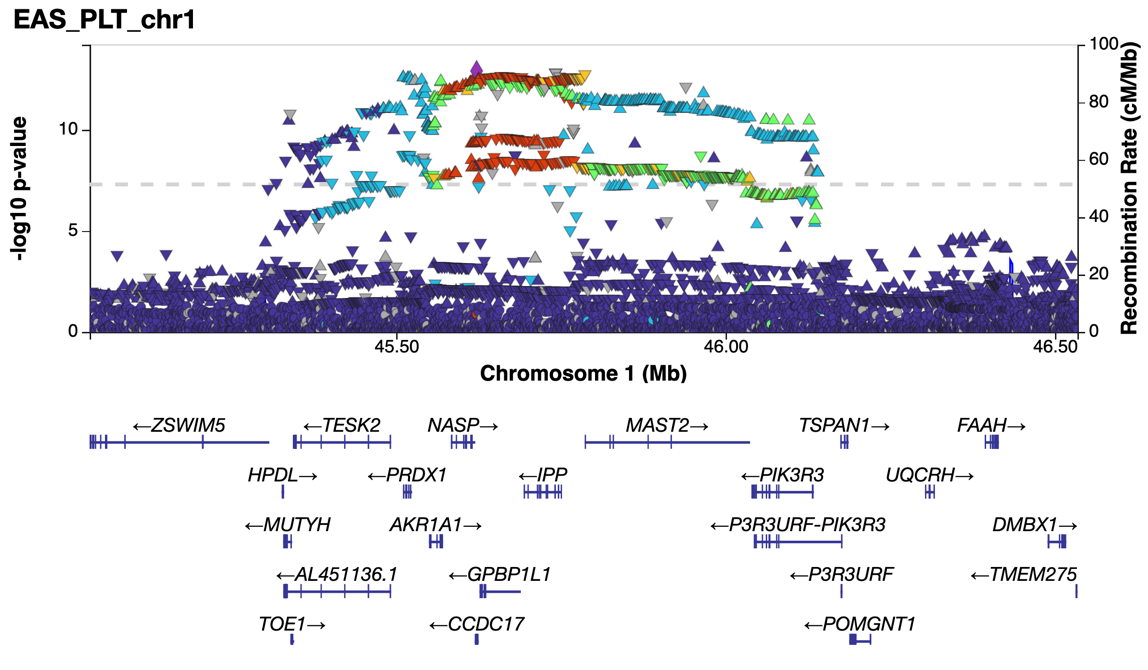


**E**

**D**

**C**

**BA**

**Figure S4: Regional plots of the locus defined by index variant rs946528.** Data are shown for **A.** Bangladeshi and **B.** Pakistani populations from this study, **C.** transancestry meta-GWAS, **D.** South Asian and **E.** East Asian ancestry-specific GWAS.

**A**

*H_4_* = 45.2%

r^2^

**B**


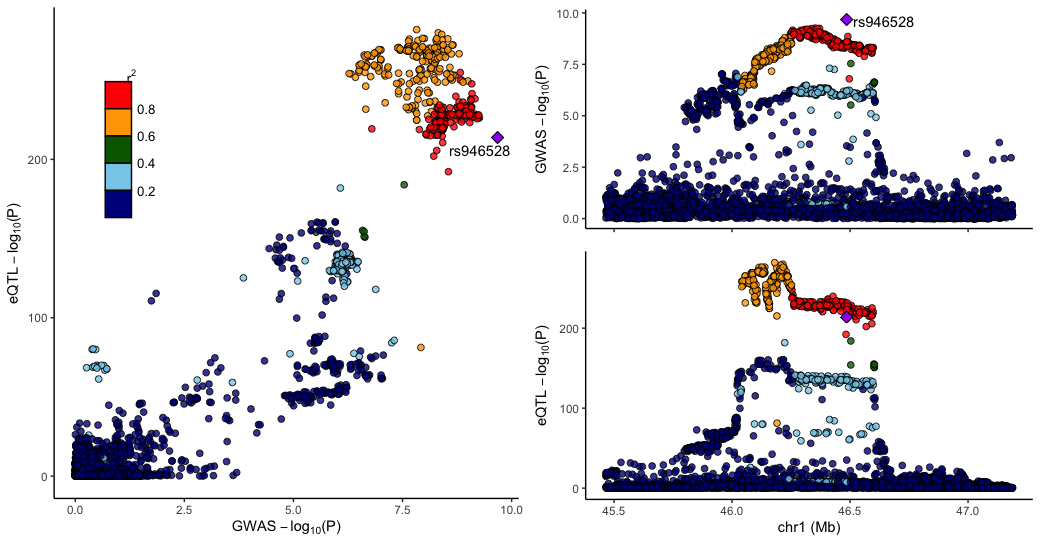


*H_4_* = 31.4%

r^2^

**Figure S5: Colocalization of variants in the r946528 locus with whole blood eQTLs for proximal genes.**

Data are presented for **A**. *MAST2* and **B**. *IPP* eQTLs. Posterior probabilities of a single causal variant (*H*_4_) were calculated using *coloc.abf()* function of R package *coloc* and plotted using *locuscomparer*. The main plots show correlation between p-values for GWAS and eQTL variants, with the inset plots showing regional views for both datasets.

**Table S1: Clinical characteristics of the study populations.** Data are reported as mean (standard deviation).

|  | **Bangladeshi (n = 20,218)** | **Pakistani (n = 9198)** |
| --- | --- | --- |
| Gender (male) | 43.4% | 41.4% |
| Age at recruitment (years) | 41.0 (12.9) | 44.6 (15.3) |
| Height (cm) | 160 (8.5) | 164 (9.1) |
| Weight (Kg) | 67.2 (12.3) | 74.4 (14.9) |
| Platelet count (x10^9^/L) | 266.4 (61.8) | 271.5 (65.1) |
| Thrombocytopenia (PLT <150 x10^9^/L)  Thrombocytosis (PLT >400 x10^9^/L) | 1.5%  2.4% | 1.8%  3.3% |

**Table S2: Allele frequencies of PLT-associated index variants from Bangladeshi analysis and in 1000 Genomes Continental Populations.** Bangladeshi index variants were defined as those with lowest p-value within each associated locus. Chromosomal positions are expressed relative to the GRCh38 genome assembly with the coded/alternate alleles on the + strand. Genes were assigned to each index variant by annotating with Variant Effect Predictor (VEP) and selecting the gene with the most severe functional consequence. BAN-Bangladeshi, PAK-Pakistani, AFR = African, AMR = Ad Mixed American, EAS = East Asian, EUR = European, SAS = South Asian**.**

| **Chromosomal position (GRCh38)** | **rsID** | **Gene(s) with VEP most severe consequence** | **Coded/alternate allele** |  | | **Superpopulation alternate allele frequency** | | | | |
| --- | --- | --- | --- | --- | --- | --- | --- | --- | --- | --- |
|  |  |  |  | **BAN** | **PAK** | **AFR** | **AMR** | **EAS** | **EUR** | **SAS** |
| chr1:46019890 | rs946528 | *MAST2* | C/T | 0.58 | 0.61 | 0.53 | 0.61 | 0.71 | 0.72 | 0.56 |
| chr1:247549001 | rs41315846 | *GCSAML* | T/C | 0.41 | 0.40 | 0.70 | 0.33 | 0.45 | 0.50 | 0.41 |
| chr2:31258101 | rs592039 | *EHD3* | G/A | 0.85 | 0.81 | 0.89 | 0.84 | 0.94 | 0.69 | 0.89 |
| chr2:159926221 | rs1877194 | Intergenic | A/G | 0.46 | 0.48 | 0.46 | 0.77 | 0.61 | 0.77 | 0.43 |
| chr3:56815721 | rs1354034 | *ARHGEF3* | T/C | 0.50 | 0.53 | 0.20 | 0.44 | 0.57 | 0.60 | 0.48 |
| chr4:6889792 | rs11734132 | Intergenic | G/C | 0.19 | 0.14 | 0.11 | 0.17 | 0.53 | 0.17 | 0.16 |
| chr4:110027510 | rs80079941 | Intergenic | G/C | 0.18 | 0.13 | 0.05 | 0.01 | 0.24 | 0.00 | 0.19 |
| chr5:66710497 | rs59596869 | *MAST4* | C/T | 0.13 | 0.06 | 0.16 | 0.01 | 0.04 | 0.01 | 0.13 |
| chr6:33575632 | rs210139 | *BAK1* | A/C | 0.71 | 0.70 | 0.52 | 0.53 | 0.73 | 0.42 | 0.74 |
| chr6:135100038 | rs34164109 | *HBS1L* | C/T | 0.11 | 0.11 | 0.14 | 0.16 | 0.24 | 0.26 | 0.11 |
| chr7:106700379 | rs342244 | Intergenic | T/G | 0.35 | 0.34 | 0.24 | 0.34 | 0.25 | 0.42 | 0.40 |
| chr8:105570896 | rs4734879 | *ZFPM2* | A/G | 0.32 | 0.33 | 0.42 | 0.31 | 0.40 | 0.29 | 0.36 |
| chr9:4788616 | rs35797651 | *RCL1* | C/G | 0.35 | 0.22 | 0.04 | 0.22 | 0.63 | 0.23 | 0.31 |
| chr9:132987359 | rs149810016 | *GFI1B* | C/A | 0.02 | 0.01 | 0.00 | 0.00 | 0.00 | 0.00 | 0.04 |
| chr10:63267383 | rs7098181 | *JMJD1C* | G/T | 0.48 | 0.48 | 0.28 | 0.30 | 0.33 | 0.43 | 0.50 |
| chr12:111411711 | rs7309325 | *SH2B3* | G/T | 0.41 | 0.35 | 0.77 | 0.32 | 0.89 | 0.20 | 0.34 |
| chr14:103098397 | rs61007561 | *EXOC3L4* | A/AG | 0.29 | 0.24 | 0.20 | 0.21 | 0.26 | 0.24 | 0.31 |
| chr17:35563315 | rs55910622 | *SLFN14* | G/T | 0.07 | 0.08 | 0.08 | 0.04 | 0.01 | 0.04 | 0.07 |
| chr18:23141009 | rs11082304 | *CABLES1* | G/T | 0.46 | 0.49 | 0.21 | 0.36 | 0.53 | 0.51 | 0.46 |
| chr20:58999408 | rs163787 | *CTSZ* | A/G | 0.80 | 0.80 | 0.88 | 0.78 | 0.86 | 0.82 | 0.84 |

**Table S3: PLT-associated index variants identified in the Pakistani population (n = 9198).** Pakistani index variants were defined as those with lowest p-value within each associated locus. Chromosomal positions are expressed relative to the GRCh38 genome assembly with the coded/alternate alleles on the + strand. Protein-coding genes were assigned to each index variant by annotating with Variant Effect Predictor (VEP) and selecting the gene with the most severe functional consequence.

| **Chromosomal position (GRCh39)** | **rsID** | **Gene(s) with VEP most severe consequence** | **Coded/**  **alternate allele** | **Alternate allele frequency** | **Beta**  **(SE)** | **p value** |
| --- | --- | --- | --- | --- | --- | --- |
| chr3:56815721 | rs1354034 | *ARHGEF3* | T/C | 0.53 | 0.114 (0.015) | 9.1 x10^- 15^ |
| chr6:33596519 | rs12206050 | Intergenic | A/T | 0.35 | 0.091 (0.015) | 2.6 x10^-09^ |
| chr6:135110180 | rs6920211 | Intergenic | T/C | 0.18 | 0.130 (0.023) | 1.6 x10^-8^ |
| chr7:106721764 | rs342284 | Intergenic | T/C | 0.31 | -0.101 (0.016) | 9.6 x10^-10^ |
| chr20:59019629 | rs34524896 | *TUBB1* | C/T | 0.13 | -0.130 (0.022) | 4.2 x10^-9^ |

**Table S4: Variants in the Bangladeshi rs946528 association interval identified as PLT-associated loci in previous GWAS.** LD was calculated using NIH LDpop Tool with LD reference dataset from the 1000G Bengali from Bangladesh population (n = 86)).

| **Chromosomal position (GRCh38)** | **rsID** | **Ancestry** | **VEP annotation** | **LD with rs946528 (r^2^)** | **Distance to rs946528 (bp)** | **Reference** |
| --- | --- | --- | --- | --- | --- | --- |
| chr1:46019890 | rs946528 | Bangladeshi | *MAST2* (intronic) | - | - | Current study |
| chr1:45786472 | rs7540578 | EAS: Japan | *MAST2* (upstream) | 0.87 | 233,418 | 11 |
| chr1:45745675 | rs61784824 | EAS: Japan | *IPP* (intronic) | 0.66 | 274,215 | 12 |
| chr1:45621905 | rs3014242 | EAS: Japan, UK, China | *CCDC17* (missense) | 0.63 | 397,985 | 1 |

**Table S5:** **Genes present in the locus containing index variant rs946528 (chr1:45575428-46137676).** The locus is defined as the interval containing all associated variants in LD (r^2^ >0.5) with index SNP rs946528. Gene names and functional annotations are derived from NCBI Gene (www.ncbi.nlm.nih.gov/gene/). Expression data for CD34-negative, CD41-positive, CD42-positive megakaryocytes from the BLUEPRINT Epigenome Project (www.blueprint-epigenome.eu/). FPKM = fragments per kilobase of transcript per million mapped reads. The posterior probability (PP) of colocalisation between variants in the Bangladeshi PLT rs946528 locus and whole blood cis-eQTLs for each gene in the interval are calculated using eQTLGen data (https://www.eqtlgen.org/).

| **Gene** | **Encoded protein** | **Biological function** | **Gene expression in MKs (FPKM)** | **PP of shared causal variant (*H*_4_)** |
| --- | --- | --- | --- | --- |
| *NASP* | Nuclear autoantigenic sperm protein | H1 histone binding protein required for nuclear histone transport that I necessary for DNA replication and cell cycle progression. | 77.65 | 0.06% |
| *CCDC17* | Coiled-coil domain containing protein 17 | Protein of unknown function predominantly expressed in lung. | 0.53 | 10.1% |
| *GPBP1L1* | GC-rich promoter binding protein 1 like 1 | Putative DNA/RNA binding protein and predicted regulator of transcription. | 80.03 | 0.46% |
| *TMEM69* | Transmembrane Protein 69 | Predicted membrane protein of unknown function. | 32.81 | 0.009% |
| *IPP* | Intracisternal A particle-promoted polypeptide | Kelch protein family member with predicted actin binding domains. | 4.95 | 31.4% |
| *MAST2* | Microtubule associated serine/threonine Kinase 2 | Microtubule associated serine/threonine kinase. | 1.54 | 45.2% |
| *PIK3R3* | Phosphatidylinositol 3-kinase regulatory subunit gamma | Regulatory component of the phosphatidylinositol 3-kinase complex that phosphorylates phosphatidylinositol and similar compounds in multiple cellular processes. | 1.28 | 0.61% |
| *P3R3URF-PIK3R3* | P3R3URF-PIK3R3 readthrough | Naturally occurring readthrough transcription between neighbouring genes LOC110117498 and *PIK3R3*. | Unknown | N/A |

**Supplementary references**

1. Chen MH, Raffield LM, Mousas A *et al*: Trans-ethnic and Ancestry-Specific Blood-Cell Genetics in 746,667 Individuals from 5 Global Populations. *Cell* 2020; **182:** 1198-1213 e1114.

2. Das S, Forer L, Schonherr S *et al*: Next-generation genotype imputation service and methods. *Nat Genet* 2016; **48:** 1284-1287.

3. Manichaikul A, Mychaleckyj JC, Rich SS, Daly K, Sale M, Chen WM: Robust relationship inference in genome-wide association studies. *Bioinformatics* 2010; **26:** 2867-2873.

4. Loh PR, Tucker G, Bulik-Sullivan BK *et al*: Efficient Bayesian mixed-model analysis increases association power in large cohorts. *Nat Genet* 2015; **47:** 284-290.

5. Bulik-Sullivan BK, Loh PR, Finucane HK *et al*: LD Score regression distinguishes confounding from polygenicity in genome-wide association studies. *Nat Genet* 2015; **47:** 291-295.

6. Marchini J, Howie B, Myers S, McVean G, Donnelly P: A new multipoint method for genome-wide association studies by imputation of genotypes. *Nat Genet* 2007; **39:** 906-913.

7. Benner C, Spencer CC, Havulinna AS, Salomaa V, Ripatti S, Pirinen M: FINEMAP: efficient variable selection using summary data from genome-wide association studies. *Bioinformatics* 2016; **32:** 1493-1501.

8. Martens JH, Stunnenberg HG: BLUEPRINT: mapping human blood cell epigenomes. *Haematologica* 2013; **98:** 1487-1489.

9. Tijssen MR, Cvejic A, Joshi A *et al*: Genome-wide analysis of simultaneous GATA1/2, RUNX1, FLI1, and SCL binding in megakaryocytes identifies hematopoietic regulators. *Dev Cell* 2011; **20:** 597-609.

10. Vosa U, Claringbould A, Westra HJ *et al*: Large-scale cis- and trans-eQTL analyses identify thousands of genetic loci and polygenic scores that regulate blood gene expression. *Nat Genet* 2021; **53:** 1300-1310.

11. Sakaue S, Kanai M, Tanigawa Y *et al*: A cross-population atlas of genetic associations for 220 human phenotypes. *Nat Genet* 2021; **53:** 1415-1424.

12. Kanai M, Akiyama M, Takahashi A *et al*: Genetic analysis of quantitative traits in the Japanese population links cell types to complex human diseases. *Nat Genet* 2018; **50:** 390-400.
